# Distinctive signatures of pathogenic and antibiotic resistant potentials in the hadal microbiome

**DOI:** 10.1186/s40793-022-00413-5

**Published:** 2022-04-25

**Authors:** Liuqing He, Xinyu Huang, Guoqing Zhang, Ling Yuan, Enhui Shen, Lu Zhang, Xiao-Hua Zhang, Tong Zhang, Liang Tao, Feng Ju

**Affiliations:** 1 Center for Infectious Disease Research, Westlake Laboratory of Life Sciences and Biomedicine, Hangzhou, 310024 Zhejiang China; 2 Key Laboratory of Coastal Environment and Resources of Zhejiang Province, School of Engineering, Westlake University, Hangzhou, 310024 Zhejiang China; 3 Key Laboratory of Structural Biology of Zhejiang Province, School of Life Sciences, Westlake University, Hangzhou, 310024 Zhejiang China; 4 Institute of Basic Medical Sciences, Westlake Institute for Advanced Study, Hangzhou, 310024 Zhejiang China; 5 Institute of Advanced Technology, Westlake Institute for Advanced Study, Hangzhou, 310024 Zhejiang China; 6 College of Marine Life Sciences, and Institute of Evolution & Marine Biodiversity, Ocean University of China, Qingdao, 266003 Shandong China; 7 Environmental Microbiome Engineering and Biotechnology Laboratory, The University of Hong Kong, Hong Kong SAR, China

## Abstract

**Background:**

Hadal zone of the deep-sea trenches accommodates microbial life under extreme energy limitations and environmental conditions, such as low temperature, high pressure, and low organic matter down to 11,000 m below sea level. However, microbial pathogenicity, resistance, and adaptation therein remain unknown. Here we used culture-independent metagenomic approaches to explore the virulence and antibiotic resistance in the hadal microbiota of the Mariana Trench.

**Results:**

The results indicate that the 10,898 m Challenger Deep bottom sediment harbored prosperous microbiota with contrasting signatures of virulence factors and antibiotic resistance, compared with the neighboring but shallower 6038 m steep wall site and the more nearshore 5856 m Pacific basin site. Virulence genes including several famous large translocating virulence genes (e.g., botulinum neurotoxins, tetanus neurotoxin, and *Clostridium difficile* toxins) were uniquely detected in the trench bottom. However, the shallower and more nearshore site sediment had a higher abundance and richer diversity of known antibiotic resistance genes (ARGs), especially for those clinically relevant ones (e.g., *fosX*, *sul1*, and TEM-family extended-spectrum beta-lactamases), revealing resistance selection under anthropogenic stresses. Further analysis of mobilome (i.e., the collection of mobile genetic elements, MGEs) suggests horizontal gene transfer mediated by phage and integrase as the major mechanism for the evolution of Mariana Trench sediment bacteria. Notably, contig-level co-occurring and taxonomic analysis shows emerging evidence for substantial co-selection of virulence genes and ARGs in taxonomically diverse bacteria in the hadal sediment, especially for the Challenger Deep bottom where mobilized ARGs and virulence genes are favorably enriched in largely unexplored bacteria.

**Conclusions:**

This study reports the landscape of virulence factors, antibiotic resistome, and mobilome in the sediment and seawater microbiota residing hadal environment of the deepest ocean bottom on earth. Our work unravels the contrasting and unique features of virulence genes, ARGs, and MGEs in the Mariana Trench bottom, providing new insights into the eco-environmental and biological processes underlying microbial pathogenicity, resistance, and adaptative evolution in the hadal environment.

**Supplementary Information:**

The online version contains supplementary material available at 10.1186/s40793-022-00413-5.

## Introduction

Hadal zone (around 6000–11,000 m), which consists of almost exclusively plunging trenches, is the deepest part on the earth known to support microbial life. Due to the rapid technological advances in deep-sea exploration and high-throughput DNA sequencing, several hadal microbial communities in the Mariana [1–4], Puerto Rico [5], Japan [6], Yap [7], and Kermadec [8, 9] Trenches have been investigated over the past decade. These studies together showed that the hadal microbial world is compositionally diverse, populationally abundant, and biologically flourishing. Likely because of its distinct biophysical and biochemical features [1, 2, 4], as well as geographical isolation, hadal zone harbors microbial communities that significantly differ from other oceanic microbial communities in compositions: common habitats in the hadal ocean include Proteobacteria, Chloroflexi, Thaumararchaea, Gemmatimonadetes, Planctomycetes, Actinobacteria, Patescibacteria, Acidobacteria, Firmicutes, and Bacterioidetes [1, 4, 9].

The ocean has been recently implicated as a global reservoir of clinically relevant antibiotic-resistant genes (ARGs) [10, 11]. However, the biological features of microbial communities at hadal zones remain largely unknown. Most previous studies described the taxonomic diversity of hadal microbial populations, while only limited studies investigated functional profiles of the communities. For instance, albeit researchers are actively studying microbial samples from the hadal zone, the pathogenic and antibiotic resistant potentials of microbial populations therein have never been closely analyzed. In other words, little is known about the presence and diversity of pathogenic and resistant genes in the hadal environments and which microorganisms actually host them. In particular, recent studies show increasing evidence for anthropogenic contamination even in the deep sea down to ~ 11,000 m [12], including polychlorinated biphenyls (PCBs) [13] and methylmercury [14] bio-accumulated in the amphipod fauna, and microplastics found in the sediment [15, 16] of hadal trenches. We propose that the existence of these man-made materials in the hadal zone may signify yet-to-be-discovered anthropogenic disturbances on the local microbial communities. However, the evolutionary mechanisms by which hadal microbiota become adapted to natural and anthropogenic (e.g., contamination) stresses remain elusive.

Here, we conducted a comprehensive study on the occurrence and distribution profiles of virulence genes (VGs), ARGs, and mobile genetic elements (MGEs) in the microbial communities of hadal sediment samples collected at three divergent locations in the Mariana Trench (Additional file 1: Fig. S1), including a bottom site of the Challenger Deep (10,898 m), a steep wall site of the trench (6038 m), and an abyssal Pacific basin site (5856 m). We also included recently published metagenomes of hadal microbiota of seawater collected at different depths in the Mariana Trench for comparative analyses [17]. We found that hadal bottom sediment samples exhibit very different profiles on both VGs and ARGs compared to the other oceanic microbial samples. Notably, our metagenomic analysis showed that the sedimental sample at 10,898 m in Challenger Deep of Mariana Trench contained a few genes encoding some of the most notorious bacterial toxins including botulinum neurotoxins, tetanus neurotoxin, and large clostridial toxins, which were occasionally detected in other hadal samples. Compared to other sample sites, we found that the total abundance and diversity of ARGs in the Challenger Deep are lower, but the spectrum is even broader. To further investigate the potentials in the dissemination of ARGs through horizontal gene transfer, we examined the diversity and distribution of MGEs. Here we provide the first systematic insights into the pathogenic and antibiotic resistance potentials of the hadal microbial communities. This study broadens our view on the biological and evolutionary features of microbial populations in the hadal zone and could be instructive for future studies on pathogenic and resistant properties of hadal inhabited microbiota.

## Methods

### Trench expedition and field sampling

Sediment samples were collected from the Pacific Ocean with the SHEN KUO research/survey vessel from November to December 2018. Samples were collected using box corer (with a base area of 400 cm^2^ and a height of 25 cm) equipped for deep-sea sediments from a depth of around 21 cm below the sediment surface and subsampled using 50 mL sterile centrifuge tubes. The tubes were stored at − 80 ℃ on board until DNA extraction from the surface sediment samples. Surface sediment samples were taken from three divergent locations (Additnal file 1: Fig. S1), including a 10,898 m bottom site of the Challenger Deep (SM10898), a 6038 m site on the steep wall of the trench (SM6038), and a 5856 m Pacific abyssal plain site (SP5856). These sampling locations were chosen because they represented Northwest Pacific locations with greatly different proximities to the coasts of China and Philippines (i.e., SP5856 vs. SM6038) and the surface of seawater (i.e., SM6038 vs. SM10898), respectively.

### DNA extraction, library construction, and sequencing

The DNA of each sample was extracted from 25 g (wet weight, 65% to 74% in water content) of sediments using the PowerMax soil DNA isolation kit (QIAGEN, made in Germany), following the low biomass sample manufacturer protocol. The DNA extracts were further purified by magnetic beads (VAHTS DNA Clean Beads Cat: N411-03) and their qualities and quantities were measured using NanoDrop™ OneC (Thermo Scientific, USA).

### Metagenomic sequencing

Each purified DNA sample was used for metagenomic library construction and paired-end shotgun sequencing on the Illumina Hi-seq platform. The genomic DNA of the Challenger Deep sediment (SM10898) was repeatedly sequenced on the Illumina Hi-seq platform to maximize the sequencing depth and coverage of metagenomic DNAs. During the experiments, all the constructed metagenomic DNA libraries were prepared without the whole genome amplification step (to avoid related bias) and sequenced with a paired-end (2 × 150 bps) strategy.

### 16S rRNA gene amplicon sequencing

The primer set 338F (5′-ACTCCTACGGGAGGCAGCAG-3′) and 806R (5′-GGACTACHVGGGTWTCTAAT-3′) were used to amplify the V3–V4 regions of the 16S rRNA gene from each purified DNA sample. PCR reactions were carried out in 30 μL reactions with 15 μL of Phusion® High-Fidelity PCR Master Mix (New England Biolabs); 0.2 μM of forward and reverse primers, and about 10 ng template DNA. The amplicon sequencing libraries were generated using Ion Plus Fragment Library Kit 48 xns (Thermo Scientific) following the manufacturer’s instructions, and sequencing was performed on the Ion S5TM XL platform. The metagenomic and amplicon sequence data are deposited in the China National GeneBank DataBase (CNGBdb) database under the accession ID: CNP0001961 (Additional file 2: Dataset S1).

### qPCR analysis of microbial biomass

To quantify the microbial biomass in the sediment samples, TaqMan qPCR with the primer set 349F (AGGCAGCAGTDRGGAAT) and 806R (GGACTACYVGGGTATCTAAT), and a TaqMan probe (FAM-TGCCAGCAGCCGCGGTAATACRDAG-TAMRA) were used to detect the V3 & V4 regions of the 16S rRNA gene. PCR reactions were carried out with 2 ng/μL template DNA using Premix Ex Taq™ Probe qPCR Kit (Takara, RR390B). The bacterial biomass of each sediment sample was calculated as 16S-copies per gram of dry biosolids (determined based on the sediment water loss after drying at 105 ℃ for 8 h).

### Functional annotation

Functional analysis of microbial communities of hadal sediment and seawater was performed using shotgun metagenomic sequence data. The raw reads were first trimmed to remove the first ten bases with low-quality using cutadapt [18], after which the quality profiles (e.g., per base sequence quality and sequence quality scores) of the clean data obtained were then validated by FastQC (v0.11.8, https://www.bioinformatics.babraham.ac.uk/projects/fastqc/). The generated clean reads were assembled using MEGAHIT (v1.2.6) [19] and the quality of assembled dataset was checked using QUAST (v5.0.2) [20]. Gene prediction was conducted using Prodigal (v2.60) in metagenome mode [21]. The predicted functions of the open reading frames (ORFs) were annotated and the pathogenicity-related factors (i.e., VGs, ARGs, and MGEs) were identified using the pipelines below.

*Virulence genes (VGs)* Functional annotation of VGs were conducted according to the Virulence Factor Database (VFDB) [22, 23], an integrated and comprehensive online resource for curating information about virulence factors of bacterial pathogens. The ORFs were searched against the extracted reference sequences of VGs using NCBI’s BLASTP with an e-value cutoff of 1e−05. A minimum amino acid identity value of 30% [24] was applied to increase the probability of capturing the real functional VGs. Further, reference sequences of human pathogenic exotoxin genes were collected from the Database of Bacterial Exotoxins for Human (DBETH) [25] which contains sequence, structure, interaction network, and analytical results for 229 toxins categorized within 24 mechanistic and activity types from 26 bacterial genera. The ORFs were searched against the extracted reference sequences of exotoxin genes using BLASTP with an e-value cutoff of 1e−05. An identity value of 30% was used for exotoxin to increase the possibility of capturing the real functional proteins.

*Antibiotic resistance genes (ARGs)* Functional annotation of ARGs were performed by two rounds of hmmscan search against Hidden Markov Models (HMMs) database. The first round of hmmscan (HMMER3) search of ORFs with the option ‘‘-cutga’’ against a self-constructed HMMs subdatabase of ARGs (i.e., ARGfams) which was built from Pfam (v 30.0) and TIGRFAMs, based on string match in their functional annotations to one of the keywords (Additional file 2: Dataset S2). Then, ARG-like ORFs were extracted and re-annotated by running 2nd round of hmmscan search with the option ‘‘-cutga’’ against the concatenated HMM database to eliminate false-positive alignments. For ARG-like ORFs with domain bit-score of best hits greater than 50 and the same annotation results in two rounds were finalized as an ARG, while those with inconsistent annotation results were manually checked to decide whether their annotation supporting their finalization as ARG or not. The identified ARGs were then classified into 12 types, including aminoglycoside, beta-lactam, bleomycin, chloramphenicol, daunorubicin, macrolide-lincosamide-streptogramin (MLS), multidrug, quinolone, tetracycline, trimethoprim, vancomycin, unclassified, followed by further classification  into 187 subtypes (Additional file 2: Dataset S3).

The diversity and abundance of ARGs were annotated with ARGs-OAP v2.0 [26], which utilizes the Structured Antibiotic Resistance Genes (SARG) database [26] to simultaneously quantify 1208 subtypes (i.e., genotype) of ARGs conferring resistance to 24 types (i.e., classes) of antibiotics. In brief, a UBLAST algorithm was first used to pre-screen ARG-like reads and 16S rRNA gene reads. Th ARG-like reads were then matched against the database using BLASTX. The reads that met the BLASTX criteria (alignment length: 25 amino acids, similarity 80%, and e-value 1e−07) were classified according to the SARG. The 16S rRNA gene-normalized relative abundance of ARGs in the metagenomic data was reported as ‘gene copy per 16S rRNA gene copy (GP16S)’ [27].

*Mobile genetic elements (MGEs)* Functional annotation of MGE genes were conducted by running the first round of hmmscan (HMMER3) search [28] of ORFs with the option ‘‘-cutga’’ against self-constructed Hidden Markov Models (HMMs) sub-database MGEfams (https://github.com/emblab-westlake/MGEfams) consisting of  MGE models extracted from Pfam (v 30.0) [29] and TIGRFAMs [30], based on string match in their functional annotations to one of the following keywords: transposase, transposon, conjugative, integrase, integron, recombinase, resolvase, conjugal, mobilization, recombination, and plasmid, as recommended previously (Forsberg et al. 2012, Forsberg et al. 2014). Afterward, MGE-like ORFs were extracted and re-annotated by running 2^nd^ round of hmmscan search with the option ‘‘-cutga’’ against the concatenated HMM database of Pfam and TIGRFAMs. For MGE-like ORFs with domain bit-score of best hits greater than 50 and the same annotation results in two rounds were finalized as an MGE, while those with inconsistent annotation results were manually checked to decide whether their annotation supporting their finalization as MGE or not. We further classified all the MGEs identified into 6 types (i.e., integrase, resolvase, plasmid, recombinase, conjugation, transposase) and 179 subtypes (Additional file 2: Dataset S4) based on the above keywords and the names of gene or gene family in the Pfam, respectively.

*Relative quantification* For each metagenome-assembled gene, their relative abundance was computed as Reads Per Kilobase (RPK). This metric was used to compare the metagenomic abundance of co-occurring ARGs and VGs detected on the same metagenome-assembled contigs.

1 $$RPK = \frac{{\sum N_{nucleobase\,mapped } / L_{ read\,length} }}{{L _{gene\,length} / 1000}}$$

where $\sum N_{nucleobase\,mapped }$: The number of all nucleobases mapped to the gene; $L_{ read\,length}$: The length of the read; $L_{ gene\,length}$: The length of the gene.

### Taxonomic analysis

*16S rRNA gene reads* The raw 16S rRNA gene amplicon sequence data were processed with the pipeline of QIIME2 [31]. Features were clustered at a 99% similarity level and taxonomically assigned against the SILVA SSU 132 database.

*Metagenomic reads* Taxonomic assignment of metagenomic reads were performed in Kraken2 package [32] and improved in Bracken package [33]. To verify the taxonomic assignment result, we also used BBMap [34] to profile bacterial community composition. In brief, metagenomic reads of each sample were mapped onto the reference sequences of the SILVA SSU Ref NR99 database (release 132) using the BBMap with 97% identity. The sequence coverage, which is the total length of mapped reads divided by the reference sequence length, was determined. The relative abundance of each taxon was calculated from the sequence coverage of each taxon (E) and the total sequence coverage (T) as E/T × 100%.

*Metagenome-assembled contigs* Taxonomic assignment of assembled contigs was based on each predicted ORF annotation result of contigs using CAT [35]. Briefly, each predicted ORF on a contig is queried with DIAMOND [36] to the NCBI non-redundant (nr) protein database. According to the annotation results, the lowest common ancestor was identified for all protein-coding genes. The taxonomic classification of the contig was jointly voted by all the ORFs on each contig, as implemented in CAT [35].

## Results and discussion

### The Challenger Deep bottom microbiota is highly prosperous

To estimate sedimental microbiota diversity from different geographical locations in the Mariana Trench, shotgun metagenome sequencing and 16S rRNA gene analyses were performed on the sedimental microbial communities from three sampling sites including the bottom of the Challenger Deep of Mariana Trench at 10,898 m (sample Name: SM10898), a steep wall site at 6038 m (SM6038), and a Pacific abyssal plain site at 5856 m (SP5856).

**Fig. 1:**
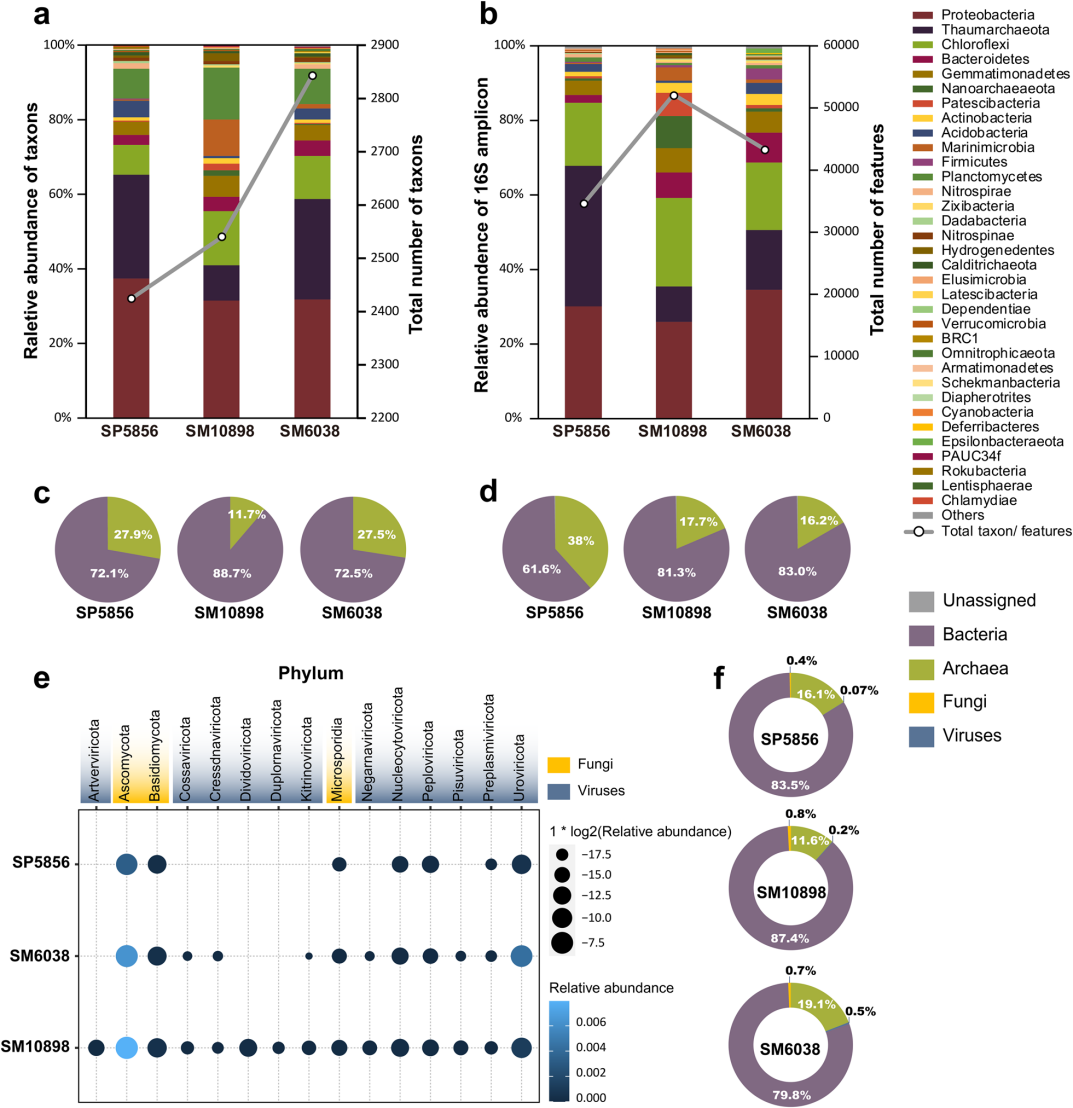
Microbial community composition in the abyssal plain and Mariana Trench sediment. **a** and **c** Bacterial and archaeal composition at the phylum (**a** top 24 across all samples shown) and kingdom (**c**) levels based on the analysis of 16S rRNA gene reads in the sediment metagenomes. **b** and **d** Bacterial and archaeal composition at the phylum (**b**) and kingdom (**d**) levels based on 16S rRNA gene amplicon sequence analysis. **e** and **f** Phylum-level fungal and viral composition (**e**, dot size scaled proportionally to relative abundance) and kingdom-level microbial composition (**f**) based on Kraken2 taxonomy assignment of the sediment metagenomes

Based on quantitative analysis of the 16S rRNA gene, the estimated bacterial biomass in the sediment samples SM10898, SM6038, and SP5856 were 1.67 × 10^7^, 9.37 × 10^6^, and 1.34 × 10^7^ 16S-copies per gram of dry biosolids. The microbial community seems to be more prosperous in the bottom of the Challenger Deep (SM10898) than in the steep wall of the trench (SM6038) and the Pacific abyssal plain (SP5856), possibly because the funnel-shaped steep-walled geomorphology of the trench allows the accumulation of rich organic compounds from various sources such as dead aquatic animals and plants, sunken terrestrial substance, and fallen sedimental matter from occasional landslides [1, 2, 37]. Accordingly, 52,092 features belong to 47 bacterial and archaeal phyla were identified from SM10898, while 43,481 features and 34,582 features of bacterial and archaeal phyla were identified from SM6038 and SP5856, respectively (Fig. 1b).

Taxonomic profiling based on 16S rRNA genes sequences showed that bacterial populations showed higher relative abundance in the hadal zone (SM10898: 81.3% from 40 phyla and SM6038: 83.0% from 41 phyla) than in the abyssal plain (SP5856: 61.6% from 36 phyla) (Fig. 1d). Proteobacteria, Chloroflexi, Thaumarchaeota, Bacteroidetes, Gemmatimonadetes, Acidobacteria, and Actinobacteria were predominant microbial organisms found in the hadal sediments (Fig. 1b), which was in line with previous studies on the microbial community composition in the Challenger Deep [2, 4, 38]. To verify the taxonomic results of 16S rRNA gene amplicon sequence analysis, taxonomic classification was also applied to the 16S rRNA gene reads in the metagenomic data of each sediment sample. The result showed a broadly similar microbial community compositional profile in which bacteria are more dominant in the hadal zone sediment (Fig. 1c). The dominant bacterial and archaeal phyla detected based on metagenomic 16S rRNA gene reads are consistent with those detected by 16S rRNA gene amplicon sequence analysis (see Fig. 1a against 1b).

To explore microbial diversity in life domains other than bacteria and archaea, we also performed the taxonomy assignment in Kraken2 and Bracken classifier which were designed for professional taxonomic classification using exact k-mer matches and the lowest common ancestor (LCA) algorithm. The classification result indicates that hadal zone (SM10898: 0.8% fungi, 0.2% viruses and SM6038: 0.7% fungi, 0.5% viruses) contain more fungi and viruses than abyssal plain (Fig. 1f). Intriguingly, while 12 and 9 virus phyla are revealed from the Challenger Deep bottom sediment (SM10898) and SM6038, sediment from the abyssal plain only contains 3 virus phyla (Fig. 1e). This result indicates that compared to shallower locations, viruses are more diverse in the hadal zone.

### The Challenger Deep hadal microbiota shows contrasting profiles of virulence genes

To comprehensively assess the pathogenic potentials of the hadal sediments, we next investigated the genetic profile of these microbial communities, particularly the virulence genes and antibiotic resistance genes (ARGs). To achieve it, we performed shotgun metagenome sequencing on the genomic DNA isolated from SM10898, SM6038, and SP5856. We also included the hadal water metagenomes in the Challenger Deep from a study by Huang et al. and the other two sediment metagenomes from the Challenger Deep [39]. General bioinformatic strategies for toxin identification by detecting sequence homology to known toxins [40] were adopted to establish the virulence gene signature profile. We constructed query sequences from the hadal microbial metagenomes and then used the alignment-based model to identify toxin homologs based on DBETH, a database consists of the vast majority of known bacterial exotoxins [25].

**Fig. 2:**
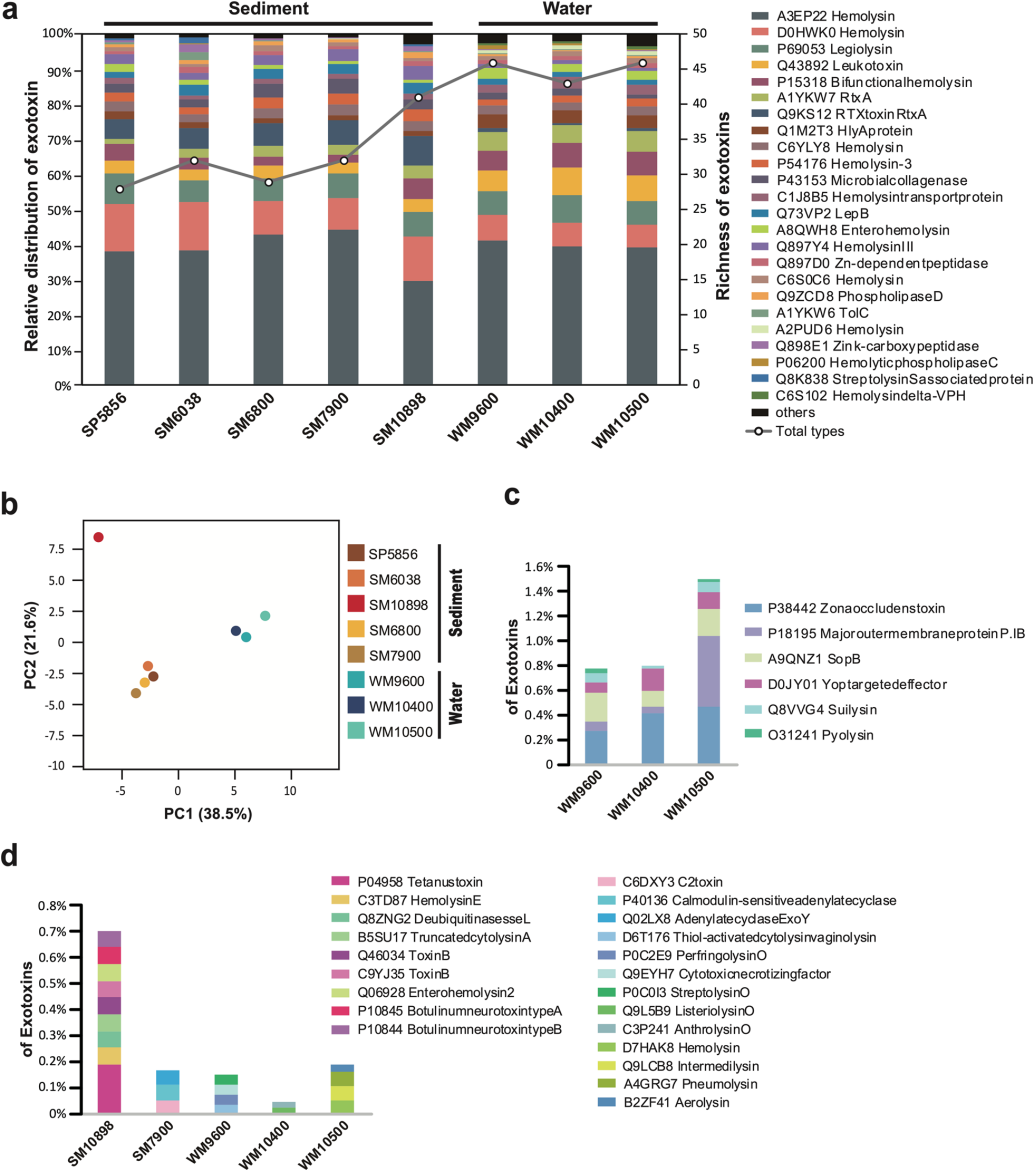
Relative abundance and richness of exotoxin genes in the microbiome of the Mariana Trench and Pacific basin sites. The bar charts and all left Y axis showed the relative distribution of exotoxin genes (**a**, **c** and **d**), while the right Y axis (**a**) denoted the richness of exotoxin genes. SP5856 represented Sediment metagenome of a 5856 m Pacific basin site (SP) near to the Mariana Trench. SM6038 and SM10898 represented Sediment metagenomes of Mariana Trench (SM) from different depths. WM9600, WM10400 and WM10500 represented Water metagenomes of Mariana Trench (WM) from different depths. **a** 24 exotoxins with the highest abundance in these samples. **b** Principal component analysis of relative abundance of exotoxin genes. % variance explained shown in parentheses. Toxin genes only shared in water are shown in **c**. Some special toxins only existing in one specific sample are shown in **d**

Most of the virulence genes found are commonly shared by all hadal metagenomes including genes encoding several hemolysins and repeats-in-toxin (RTX) proteins (Fig. 2a; Additional file 2: Dataset S5). Interestingly, there is a clear compositional distinction of virulence gene profiles between the water microbiota and sedimental microbiota in the hadal zone (Fig. 2b). Some exotoxin genes got enriched in sedimental microbiota (Additnal file 1: Fig. S2a and S2b), while some others were more abundant in water microbiota (Additnal file 1: Fig. S2c and S2d). There were ~ 1% of virulence genes only shared in water samples (Fig. 2c), but very few were limited to sedimental samples. Notably, the virulence gene profile of the Challenger Deep bottom sediment (SM10898) is particularly intriguing, as it largely differs from others and contains enriched genes encoding several famous large bacterial toxins including botulinum neurotoxins, tetanus neurotoxin, and clostridial glucosylating toxins (Fig. 2b). In contrast, we did not observe any related genes in all the other hadal microbial communities examined (Fig. 2d). These results indicate potential selective enrichment of botulinum/tetanus neurotoxins and large clostridial toxin genes at the Challenger Deep bottom. Future research efforts are needed to resolve the underlying selective pressure of these most potent toxin genes in this extreme hadal environment.

We further identified general virulence factors, including bacterial toxins, cell surface proteins that mediate the bacterial attachment, cell surface carbohydrates and proteins that protect a bacterium, and hydrolytic enzymes that may contribute to the pathogenicity of the bacterium. We observed a much higher number of VFG types in the water metagenomes than sediment metagenomes, while a unique spectrum of virulence factors was noticeable in the deepest sediment (SM10898, Additnal file 1: Fig. S3, Additional file 2: Dataset S6).

### The Challenger Deep hadal microbiota harbors diverse antibiotic resistome

**Fig. 3:**
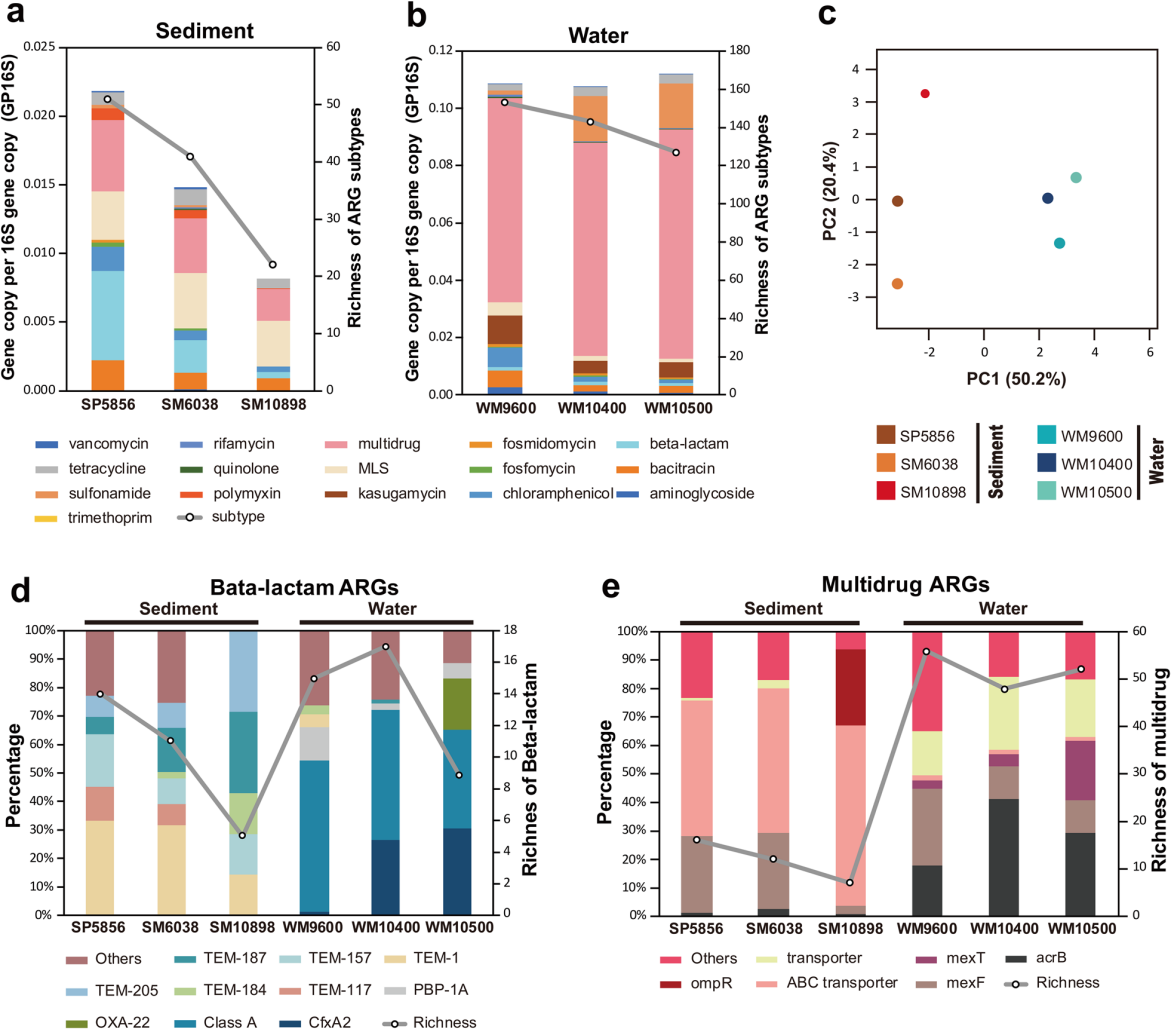
Relative abundance and richness of antibiotic resistance genes (ARGs, **a** and **b**) in the sediment and seawater microbiome of the Mariana Trench. The left Y axis and bar charts showed the relative abundance of ARGs (**a** and **b**) or relative percentage of ARG subtypes (**d** and **e**), while the right Y axis and lines denoted the richness of ARG subtypes (**a** and **b**) or the richness of beta-lactam and multidrug (**d** and **e**). **c** Principal component analysis of relative abundance of ARGs, % variance explained shown in parentheses. SP5856, SM6038 and SM10898 represented sediment metagenomes from different depths. WM9600, WM10400, WM10500 represented seawater metagenomes from different depths

To explore the collection of ARGs or antibiotic resistome in the Mariana Trench, we used ARGs-OAP v2.0 [26] to analyze the clean reads of all sediment metagenomes to quantify a broad spectrum of known ARGs (Additional file 2: Dataset S7). Overall, we found a marked cross-habitat difference in the resistome composition between the sediment (a) and seawater (b) of the Mariana Trench (Fig. 3), co-driven by habitat selection, biotic interactions (e.g., species competition), and anthropogenic contamination.

*Hadal sediment resistome* Both the abundance and richness of ARGs greatly differed across sediment sites in the following increasing trend: SM10898 < SM6038 < SP5856 (Fig. 3a). Surprisingly, the bottom site of the Challenger Deep (SM10898) was found to still harbor 20 subtypes of known ARGs that accounted for a total abundance of 9.87 × 10^−3^ gene copies per 16S rRNA gene copy (GP16S), or an estimated frequency of resistance of 6.65 × 10^−3^ gene copies per cell (GPC). From the Challenger Deep to its neighboring but shallower steep wall site (SM6038), the total abundance and diversity of known ARGs were both increased by 81% (left Y, bars) and 86% (right Y, grey lines), respectively (Fig. 3a). The dramatic change was mostly contributed by the abundance increase in the ARGs of beta-lactam (TEM-1/187/91/117/75/178/157/205/146/89) and polymyxin (*arnA*), followed by fosfomycin (*fosX*), rifamycin (*ros*), aminoglycoside (*aph*(*6*)-*I*), vancomycin (*vanR*), rifamycin (*qepA*) and sulfonamide (*sul1*) resistance genes which were detected in the steep wall (2.82 × 10^−5^ to 1.37 × 10^−4^ GP16S each), but not in the Challenger Deep bottom site (Fig. 3a). However, the compositional profiles of ARGs of both trench site sediments were significantly correlated (P < 10^−5^), whether compared at the level of ARG type (R = 0.93, n = 13) or subtype (R = 0.96, n = 38), revealing considerable similarity in their resistome composition.

When the bottom site sediment of Challenger Deep (SM10898) was further compared against that of the Pacific abyssal plain (SP5856) (Fig. 3a), we observed a dramatic increase in the total ARG abundance by 167% (8.17 × 10^−3^ to 2.18 × 10^−2^ GP16S) and diversity by 140% (20–48 subtypes). This was mostly contributed by the marked increase in the ARGs of beta-lactam (TEM-1/157/117/205/178/171/187/75/188/6/91/195/124 and OXA-209, Fig. 3d and Additnal file 1: Fig. S4) and multidrug (*mexF/W/I*, *emrA*, *mdfA*, *acrB*, *ceoB*, *mdtB/H/C*, *mexD*, *oprC* and *cmeB*, Fig. 3e and Additnal file 1: Fig. S4), followed by polymyxin (arnA) and chloramphenicol (cat, floR and cmx) resistance genes (Additnal file 1: Fig. S4). Meanwhile, the compositional profile of ARG types in the bottom sediment of Challenger Deep was more similar to the shallower steep wall sediment (SM6038, R = 0.93), compared with that of abyssal plain sediment (SP5856, R = 0.67).

In summary, our data show markedly higher total abundance and richer diversity of ARGs in the sediments collected arefrom shallow (SM6038) or near shore (SP5856) locations, which hypothetically reflects the stronger anthropogenic stress (e.g., pollution) at the shallower sites compared with the 10,898 m bottom of Challenger Deep. Our hypothesis is well supported by the higher abundance and richer diversity of clinically relevant extended-spectrum beta-lactamases (e.g., TEM family, Additnal file 1: Fig. S4) and sulfonamide resistance genes (*sul1*, Additnal file 1: Fig. S4) in the neighboring but shallower (SM6038) or more nearshore site (SP5856) [41], which could be more accessible by anthropogenic contamination.

*Hadal seawater resistome* Both the abundance (1.08 to 1.12 × 10^−1^ GP16S each) and diversity (127 to 153 subtypes) of ARGs (Fig. 3b) were surprisingly high in the hadal seawater (down to ~ 10,500 m) of the Mariana trench. In other words, the hadal seawater contains an extremely high level of ARGs that is comparable to the one reported in the anthropogenically impacted wastewater and sewage sludge (10^−2^ to 10^−1^ GP16S), and exceeds that of normal drinking water, river water, soil, and ocean sediment (10^−3^ to 10^−2^ GP16S) by up to two orders of magnitude [42, 43].

Intriguingly, resistome comparison among the seawater between 9600 to 10500 m showed a sharp and almost linearized decrease in the ARG diversity whereas a slight increase in the total ARG abundance contributed by the marked enrichment (> 1 time) of sulfonamide and multidrug resistance genes (Fig. 3b). This opposite changing trend in the abundance versus diversity of ARGs complied with the strong enrichment of cAMP-regulatory protein-coding genes (3.44 × 10^−2^ GP16S) that mediates antibiotic resistance or tolerance by 4.7 times, suggesting a potential selection of antibiotic resistance in the trench bottom seawater. In turn, our discovery of the strikingly strong selective enrichment of multidrug efflux pump genes *acrB* and *mexT* (Fig. 3e), and specifically sulfonamide resistance gene *sul2* (Fig. 3b) in the seawater microorganisms below 9600 m should theoretically enable their rapid adaption to inhibitory chemicals of anthropogenic origins, such as some persistent organic pollutants (POPs) reported in the Mariana Trench bottom [13]. For instance, multidrug efflux pumps capable of exporting different classes of drugs should confer cross-resistance to an entire or multiple classes of antimicrobial metabolites or contaminates, while co-resistance to sulfonamide antibiotics can be preferentially selected through horizontal co-transfer of *sul2* with other stress-responsive genes into the same bacteria as might occur on a plasmid or be facilitated by other MGEs with vertical gradient changes below 9600 m.

*Deterministic drivers of hadal resistome* The observed resistome differences between hadal sediment and seawater and between different water depths are at least driven by deterministic natural processes including habitat (environmental) filtering and biotic interactions (e.g., competition). First, habitat filtering is a widespread process shaping bacterial community structure. Because the bacteriome structure is demonstrated to correlate with resistome composition across habitats in soil [44] and wastewater treatment plants [27], habitat filtering can thus indirectly determine resistome composition through direct effects on the bacteriome. Our argument also well explained the observed clustering of resistome composition by environmental habitat types (n = 10). The filtering effect together with the known co-selection of antibiotic resistance with changing environmental conditions (e.g., temperature [45], salinity [46, 47] and pH [48, 49]) may co-explain the observed resistome differentiation across environmental habitats and gradients of the Mariana Trench (Fig. 3a vs. 3b, c). Moreover, naturally occurring antimicrobial secondary metabolites (e.g., antibiotics) are widely acknowledged as a conditioning agent between competing microbial species in soil and ocean. Therefore, local selection and maintenance of natural ARGs in Challenger Deep bottom sediment (10,898 m) is likely contributed by local strains. For example, Actinomycetes have been reported to possess promising bioactive and biosynthetic potentials for antimicrobial metabolites production [50]. Consistent with the viewpoint, our 16S data showing that Actinomycete populations are both richer (1474 OTUs) and more abundant (2.83%) in the sediment of Challenger Deep bottom (SM10898) relative to the shallower basin site (SP5856: 457 OTUs and 1.32%).

Despite the afore-discussed role of natural processes in shaping hadal resistome, the potential co-effects of anthropogenic processes (e.g., contamination) cannot be ruled out, especially when our data first demonstrated surprising diversity and abundance of both clinically important VGs and ARGs (e.g., *fosX*, *sul1* and TEM-family extended-spectrum beta-lactamases) of potential anthropogenic sources in the Mariana trench bottom. Other evidence for an anthropogenic disturbance on the Trench environment included the recent finding of the presence and accumulation of POPs (e.g., PCBs, PBDEs, and PAHs) in the bottom sediment and fauna of the Mariana Trench [13]. Notably, the bio-toxicities and antimicrobial effects of some POPs (e.g., PAHs) are known to promote the co-selection of ARGs in soil and ocean bacteria [51–53], although it remains examined whether this could be the case in the Mariana Trench bottom habitats such as water, sediment and fauna guts. In summary, both natural and anthropogenic processes can co-determine the selection patterns and composition of hadal resistome. Efforts are needed to examine the relative contribution of natural versus anthropogenic factors and processes on the hadal microbiome assembly and resistome selection, and check whether anthropogenically derived pollutants (e.g., POPs) are reported in the trench bottom may promote local selection of antibiotic resistance and pathogenicity.

### Mobilome differentiation between hadal sediment and seawater reveals contrasting routes for horizontal gene transfer in Mariana Trench

**Fig. 4:**
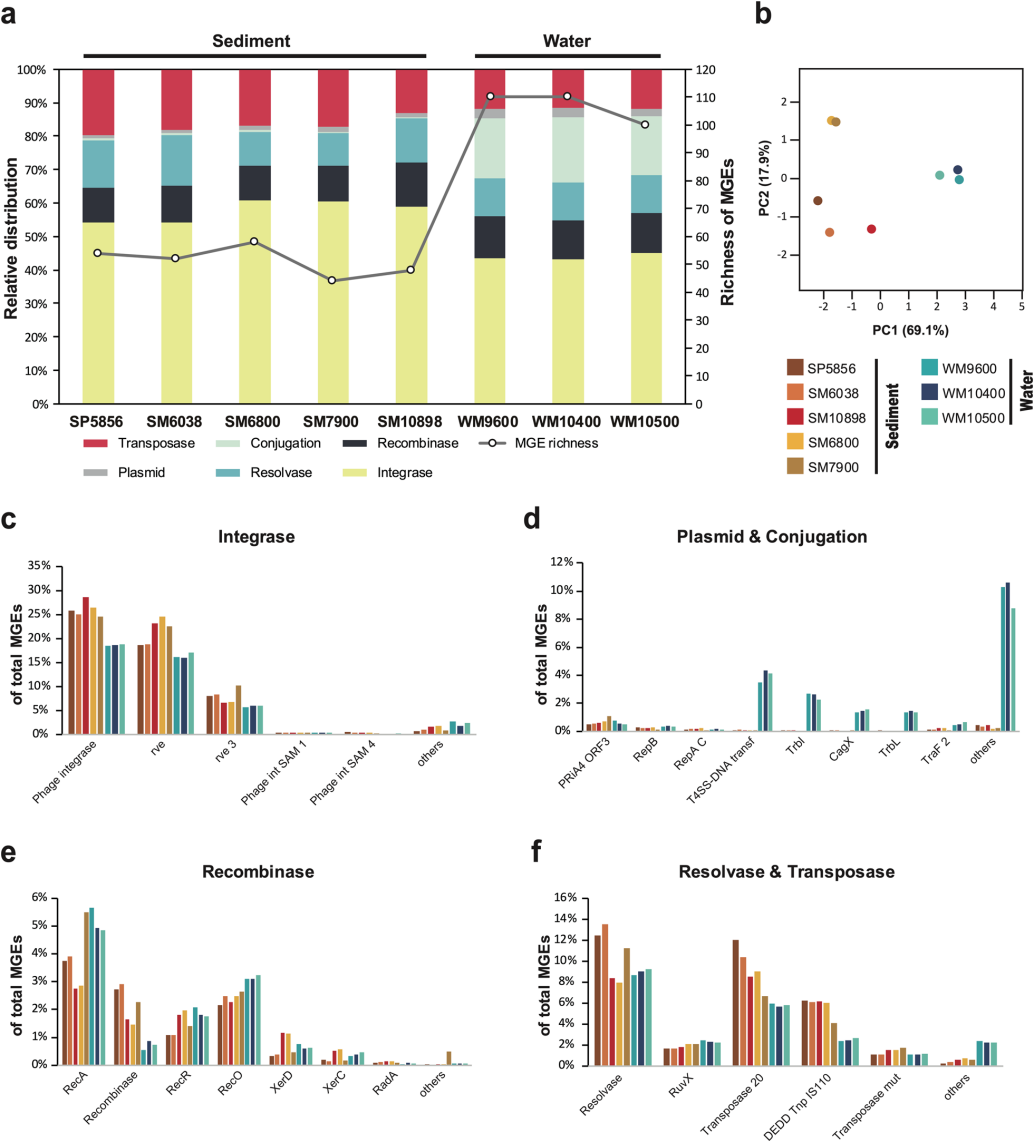
Relative distribution and richness of MGEs in the sediment and seawater microbiome of the Mariana Trench. The bar charts and all left Y axis showed the relative distribution of MGEs in the mobilome (**a–f**), while the right Y axis (**a**) denoted the richness of MGEs. **b** Principal component analysis of relative abundance of ARGs, % variance explained shown in parentheses. SP5856, SM6038, SM6800, SM7900 and SM10898 represented sediment metagenomes from different depths

The mobilome (i.e., the collection of MGEs) of the microbiome enables marine bacteria to quickly acquire novel traits to survive environmental changes through horizontal gene transfer (HGT). To check which HGT mechanisms may contribute to the hadal adaptation and the evolution of the Mariana Trench microbiota, we predicted mobility genes based on a strategic two-step hmmscan search against two concatenated HMM databases with only mobility genes and with all functional genes, respectively (Additional file 2: Dataset S8). Overall, the hadal sediment and water mobilomes showed clearly different richness (a) and composition (b) of MGEs (Fig. 4). However, both habitat mobilomes were dominated by integrase genes (54.2–60.9% of sediments, and 43.1–45.1% of seawater, Fig. 4a), especially those encoded by phage (Fig. 4c), implying the importance of phage-mediated HGT mechanisms (i.e., viral transduction) for mediating the evolution of hadal microbiome in the Mariana Trench. Viruses are the most abundant life form in the ocean with the richest genetic diversity [54]. When a virus invades and shuttles between different hosts, it has the potential to integrate new genetic materials into its hosts (or progeny virus), thereby promoting the exchange of genetic information between marine microorganisms. Therefore, marine viruses are the main driver of the evolution of both host and viral assemblages in the ocean environment.

Noticeably, conjugation- and plasmid-related MGEs accounted for a negligible relative percentage in the sediment mobilome (Fig. 4a, d), whereas they were found abundant (17.4–19.3 and 2.3–2.8% respectively) in the seawater mobilome (Fig. 4a), revealing plasmid and conjugation-mediated HGT should be much more common in the hadal seawater than sediment of the Mariana Trench. The prevalence of plasmid DNA and conjugative gene transfer requires physical contact between microbes or between microbes and plasmids. We conjecture that conjugation and plasmid-mediated HGT is less efficient in the sediment than the seawater, possibly due to the restricted dispersal of microorganisms and the lack of sufficient physical contact essential for conjugation. In the trench where biomass density is extremely low, the flowing seawater provides opportunities for physical contact of microbes, while the movement and diffusion of microbial communities are restricted in the sediment beneath the flowing water. Collectively, the hadal sediment and seawater mobilome show contrasting diversity and composition of MAEs, especially that phage- and integrase-mediated horizontal gene transfer is key mechanisms in the evolution of microorganisms in the Mariana Trench. Future efforts are needed to resolve the underlying driving force of vertical dynamics and cross-habitat differentiation of mobilome composition in the Mariana trench microbiota.

### Transferable antibiotic resistance and virulence genes are potentially co-selected in taxonomically diverse microorganisms inhabiting the Mariana Trench sediment

**Fig. 5:**
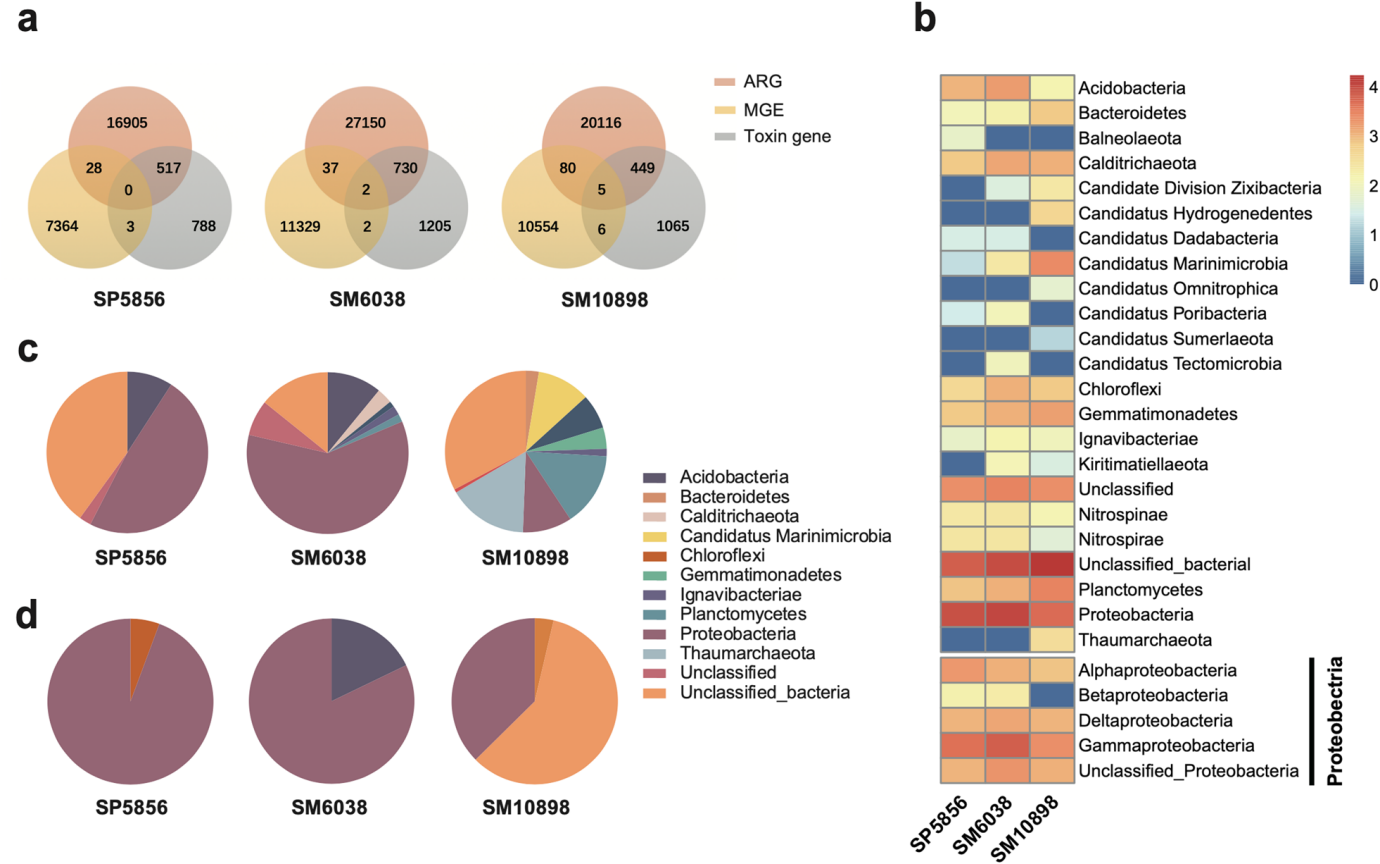
Contig-level co-occurring and taxonomic analysis of ARGs, MGEs and/or toxin genes. **a** Venn diagram showing number of contigs with co-occurring versus independently occurring gene categories. **b** Metagenomic abundance of co-occurring ARGs and toxin genes summed by phylum-level (and Class-level of Proteobacteria phylum) contig taxonomy, expressed as log_10_(RPK + 1). **c** and **d** Relative taxonomic distribution of relative abundance (RPK) with mobilized ARGs (**c**) and mobilized toxin genes (**d**)

The mobilome mediates the spread of antimicrobial resistance and virulence factors via HGT. To explore the spreading potentials of ARGs and VGs of the sediment microbiome of Mariana Trench, we compared the co-occurrence instances between ARGs (Additional file 2: Dataset S9), MGEs, and VGs on the same metagenome-assembled contigs (Fig. 5a). We first queried ORFs predicted from each contig with DIAMOND [36] against the NCBI non-redundant protein database (nr). The ORFs were then individually classified based on the LCA of the top 10% hits and their results were used for taxonomic classification of contigs with CAT [35].

Overall, we detected 517, 732, and 454 contigs with co-occurring ARGs and VGs from sediment SP5856, SM6038, and SM10898, respectively, accounting for 0.43‰ to 0.60‰ in each contig set (Fig. 5a). These VGs-carrying resistance contigs were most assigned to unclassified Bacteria (30.28–47.43%) and Proteobacteria (14.59–38.76%), followed by Planctomycetes, Gemmatimonadetes, Calditrichaeota, and Acidobacteria (Fig. 5b). In all samples, the co-occurrence between ARGs and VGs (449–730 contigs) was more frequent than the co-occurrence between ARGs and MGEs (28–80 contigs) or between MGEs and VGss (2–6 contigs), which may be the co-existence of ARGs and VGs in pathogenicity islands (PAIs). PAIs are usually located in the bacterial genomes and contribute to microbial evolution by HGT events [55]. While the Challenger Deep (SM10898) and its neighboring but shallower steep wall site of the trench (SM6038) are geographically close, the species composition and taxonomic distribution of VGs-carrying resistance contigs are different (Additnal file 1: Fig. S5). Instead, the microbial community of the ocean basin (SP5856) was relatively closer to that of SM6038. In the Challenger Deep (SM10898), more than half of the VGs-carrying resistance contigs (54.02%) could not be classified at the phylum level, while this value was only 41.17% and 39.69% in other sample sites, suggesting uncharacterized microbes containing ARGs and VGs in the deepest trench sediment.

Compared with the basin site (SP5856) and the steep wall sites (SM6038), the Challenger Deep (SM10898) harbored 7.05 and 4.67-fold of mobilized ARGs (i.e., an ARG that co-occurs with at least an MGE gene) that were taxonomically distributed in 8 prokaryotic phyla including Thaumarchaeota, Planctomycetes, Candidatus Marinimicrobia, Proteobacteria, Chloroflexi, Gemmatimonadetes, Bacteroidetes, and Ignavibacteriae (Fig. 5c). Likewise, mobilized VGs were also more abundant (3.6-fold) in the Challenger Deep (1888.40 RPK) compared to the ocean basin site (SP5856: 521.89 RPK) and the steep wall site (SM6038: 517.59 RPK), albeit ~ 59% of these bacterial genes failed to be effectively classified (Fig. 5d). These results revealed that the extreme environment of the trench bottom may enhance the propagation of mobilized ARGs and VGs, which originated with frequent HGT events and would reflect long-term adaptive traits of microorganisms inhabiting the bottom sediment of the Challenger Deep.

## Conclusions

This study depicts the landscape of virulence factors, antibiotic resistome, and mobilome of the microbial community in the hadal zone of the Mariana Trench. The results generally showed the deepest hadal sediment harbored much higher abundance and more types of known exotoxins than sediments from shallower locations. Both these VGs and ARGs showed clearly distinct diversity and distribution patterns between hadal sediments and waters. Notably, clinically relevant virulence genes and ARGs were highly diverse in the bottom seawater (below 9600 m) of Mariana Trench, accompanied by a strong selection of sulfonamide resistance and multidrug resistance genes which deserves further investigation. In summary, our study provides the first systematic overview of distribution patterns and diversity of pathogenicity, resistance, and mobility factors of the Mariana Trench microbiome. Our new findings not only provide new evidence for further analysis of the evolutionary process of microbial pathogenicity, resistance, and HGT in the extreme hadal environment but also puts forward an open question on the diversity and relative contribution of selective pressure of natural and anthropogenic origins on the local development and spread of antibiotic resistance and microbial pathogenicity in the deepest part of the Earth’s surface.

## Supplementary Information

**Additional file 1. Fig. S1**: Sampling point on Pacific Ocean. **Fig. S2**: Exotoxin genes of which different richness were observed in the microbiome of the Mariana Trench and Pacific basin sites. (a) and (b) show exotoxin genes which exist higher abundance in sediment than in water. Similarly, (c) and (d) show exotoxins which are more present in water. **Fig. S3**: Relative abundance of virulence factor genes in the microbiome of the Mariana Trench and adjacent basin. 50 virulence factors with the highest abundance in these metagenomes were shown in (a). Color of each box is correlated with the relative abundance of each virulence factor in samples. (b) shows number of type of found virulence factors in each metagenome. **Fig. S4**: Relative abundance and richness of ARGs in the sediment microbiome of the Mariana Trench. The left Y axis and bar charts showed the relative percentage of ARG subtypes while the right Y axis and lines denoted the abundance sum (as copies per 16S rRNA gene copy, GP16S, b, c and d) of ARG subtypes. SP5856, SM6038 and SM10898 represented sediment metagenomes from different depths. The symbols before gene names, i.e., *, ** and !, denoted the degrees of fold change in abundance from SM10898 to SP5856 of ≥ 1, ≥ 10 and positive infinity, respectively. **Fig. S5**: Relative distribution of the taxonomy (in phylum-level) of toxin gene-carrying resistance contigs taxonomy. **Fig. S6**: Principal component analysis of relative abundance of ARGs which was annotation by predicted ORFs, % variance explained shown in parentheses. SP5856, SM6038 and SM10898 represented sediment metagenomes from different depths. WM9600, WM10400, WM10500 represented seawater metagenomes from different depths.

**Additional file 2. Datasets S1**: metagenomic data information. **Dataset S2**: key words which were used to extract the hmm models to construct the ARGfams from Pfam (v 30.0) and TIGRFAMs. **Dataset S3**: ARG types and subtypes' information of domain based annotation result. **Dataset S4**: MGE types and subtypes' information. **Dataset S5**: The number of toxin genes predicted from the sediment metagenomes based on DBETH. **Dataset S6**: The number of toxin genes predicted from the sediment metagenomes based on VFDB. **Dataset S7**: Relative abundance of known ARGs in the sediment metagenomes based on ARGs-OAP v2.0 pipeline analysis. Unit: copies per 16S rRNA gene copy (GP16S). **Dataset S8**: Number and distribution of MGEs genes predicted from the sediment metagenomes based on MGEfams. **Dataset S9**: Number of ARGs predicted from the sediment metagenomes based on ARGfams.

## Data Availability

The metagenomic and amplicon sequence data are deposited and publicly available in the China National GeneBank DataBase (CNGBdb) database under the accession ID: CNP0001961 via the following link: https://db.cngb.org/search/project/CNP0001961/.
