# Supplementary material for: Distinctive signatures of pathogenic and antibiotic resistant potentials in the hadal microbiome
Source: Environ Microbiome. 2022 Apr 25;17:19. doi: 10.1186/s40793-022-00413-5 (PMC9036809; doi:10.1186/s40793-022-00413-5)
Supplement: Supplementary file 1 — Additional file 1. Fig. S1: Sampling point on Pacific Ocean. Fig. S2: Exotoxin genes of which different richness were observed in the microbiome of the Mariana Trench and Pacific basin sites. (a) and (b) show exotoxin genes which exist higher abundance in sediment than in water. Similarly, (c) and (d) show exotoxins which are more present in water. Fig. S3: Relative abundance of virulence factor genes in the microbiome of the Mariana Trench and adjacent basin. 50 virulence factors with the highest abundance in these metagenomes were shown in (a). Color of each box is correlated with the relative abundance of each virulence factor in samples. (b) shows number of type of found virulence factors in each metagenome. Fig. S4: Relative abundance and richness of ARGs in the sediment microbiome of the Mariana Trench. The left Y axis and bar charts showed the relative percentage of ARG subtypes while the right Y axis and lines denoted the abundance sum (as copies per 16S rRNA gene copy, GP16S, b, c and d) of ARG subtypes. SP5856, SM6038 and SM10898 represented sediment metagenomes from different depths. The symbols before gene names, i.e., *, ** and !, denoted the degrees of fold change in abundance from SM10898 to SP5856 of ≥ 1, ≥ 10 and positive infinity, respectively. Fig. S5: Relative distribution of the taxonomy (in phylum-level) of toxin gene-carrying resistance contigs taxonomy. Fig. S6: Principal component analysis of relative abundance of ARGs which was annotation by predicted ORFs, % variance explained shown in parentheses. SP5856, SM6038 and SM10898 represented sediment metagenomes from different depths. WM9600, WM10400, WM10500 represented seawater metagenomes from different depths. [file 40793_2022_413_MOESM1_ESM.docx]

SUPPLEMENTARY MATERIAL **for**

Distinctive signatures of pathogenic and antibiotic resistant potentials in the hadal microbiome

Liuqing He^#^, Xinyu Huang^#^, Guoqing Zhang, Ling Yuan, Enhui Shen, Lu Zhang, Xiao-Hua Zhang, Tong Zhang, Liang Tao*, Feng Ju*

Westlake University，China

*Corresponding author:

Liang Tao, Ph.D. Telephone: +86-571-86929603; Email: [taoliang@westlake.edu.cn](mailto:taoliang@westlake.edu.cn) ;

Feng Ju, Ph.D. Telephone: +86-571-87963205; Email: jufeng@westlake.edu.cn

**Figures**

**Figure S1**. Sampling point on Pacific Ocean

**Figure S2.** Exotoxin genes of which different richness were observed in the microbiome of the Mariana Trench and Pacific basin sites. (a) and (b) show exotoxin genes which exist higher abundance in sediment than in water. Similarly, (c) and (d) show exotoxins which are more present in water.

**Figure S3.** Relative abundance of virulence factor genes in the microbiome of the Mariana Trench and adjacent basin. 50 virulence factors with the highest abundance in these metagenomes were shown in (a). Color of each box is correlated with the relative abundance of each virulence factor in samples. (b) shows number of type of found virulence factors in each metagenome.

**Figure S4.** Relative abundance and richness of ARGs in the sediment microbiome of the Mariana Trench. The left Y axis and bar charts showed the relative percentage of ARG subtypes while the right Y axis and lines denoted the abundance sum (as copies per 16S rRNA gene copy, GP16S, b, c and d) of ARG subtypes. SP5856, SM6038 and SM10898 represented sediment metagenomes from different depths. The symbols before gene names, i.e., *, ** and !, denoted the degrees of fold change in abundance from SM10898 to SP5856 of ≥1, ≥10 and positive infinity, respectively.

**Figure S5.** Relative distribution of the taxonomy (in phylum-level) of toxin gene-carrying resistance contigs taxonomy.

**Figure S6.** Principal component analysis of relative abundance of ARGs which was annotation by predicted ORFs, % variance explained shown in parentheses. SP5856, SM6038 and SM10898 represented sediment metagenomes from different depths. WM9600, WM10400, WM10500 represented seawater metagenomes from different depths.


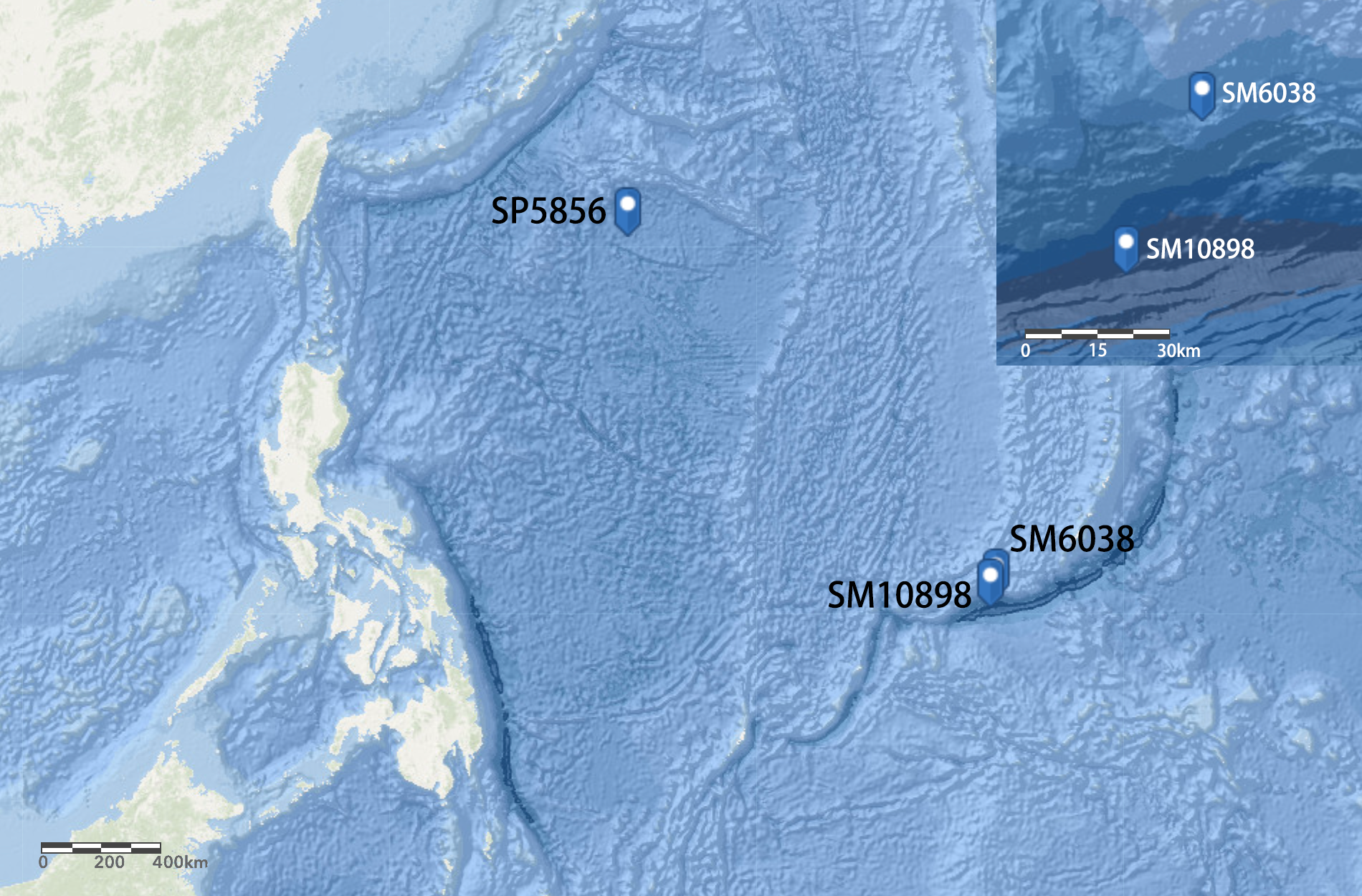


**Figure S1.** Sampling point on Pacific Ocean


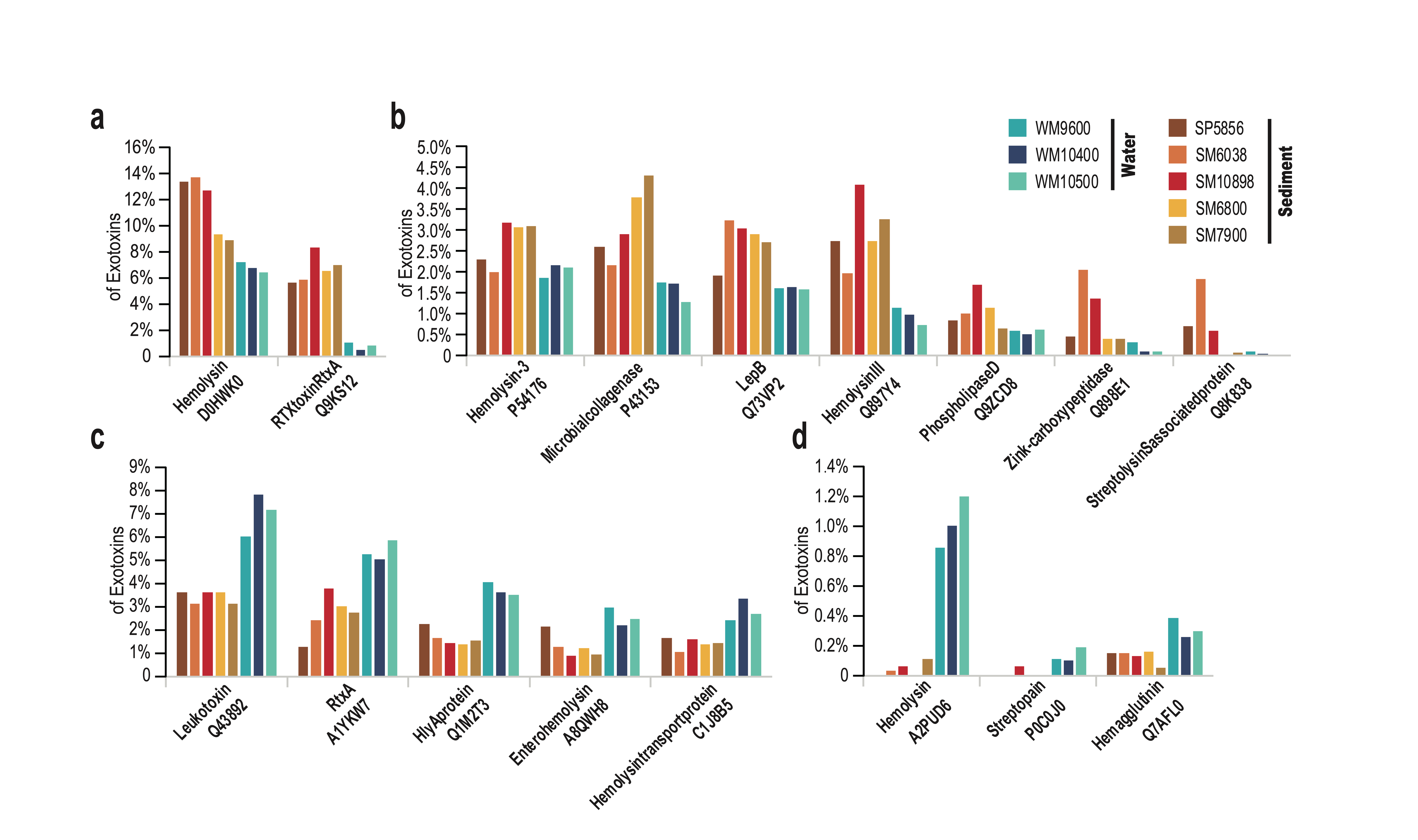


**Figure S2.** Exotoxin genes of which different richness were observed in the microbiome of the Mariana Trench and Pacific basin sites. (a) and (b) show exotoxin genes which exist higher abundance in sediment than in water. Similarly, (c) and (d) show exotoxins which are more present in water.


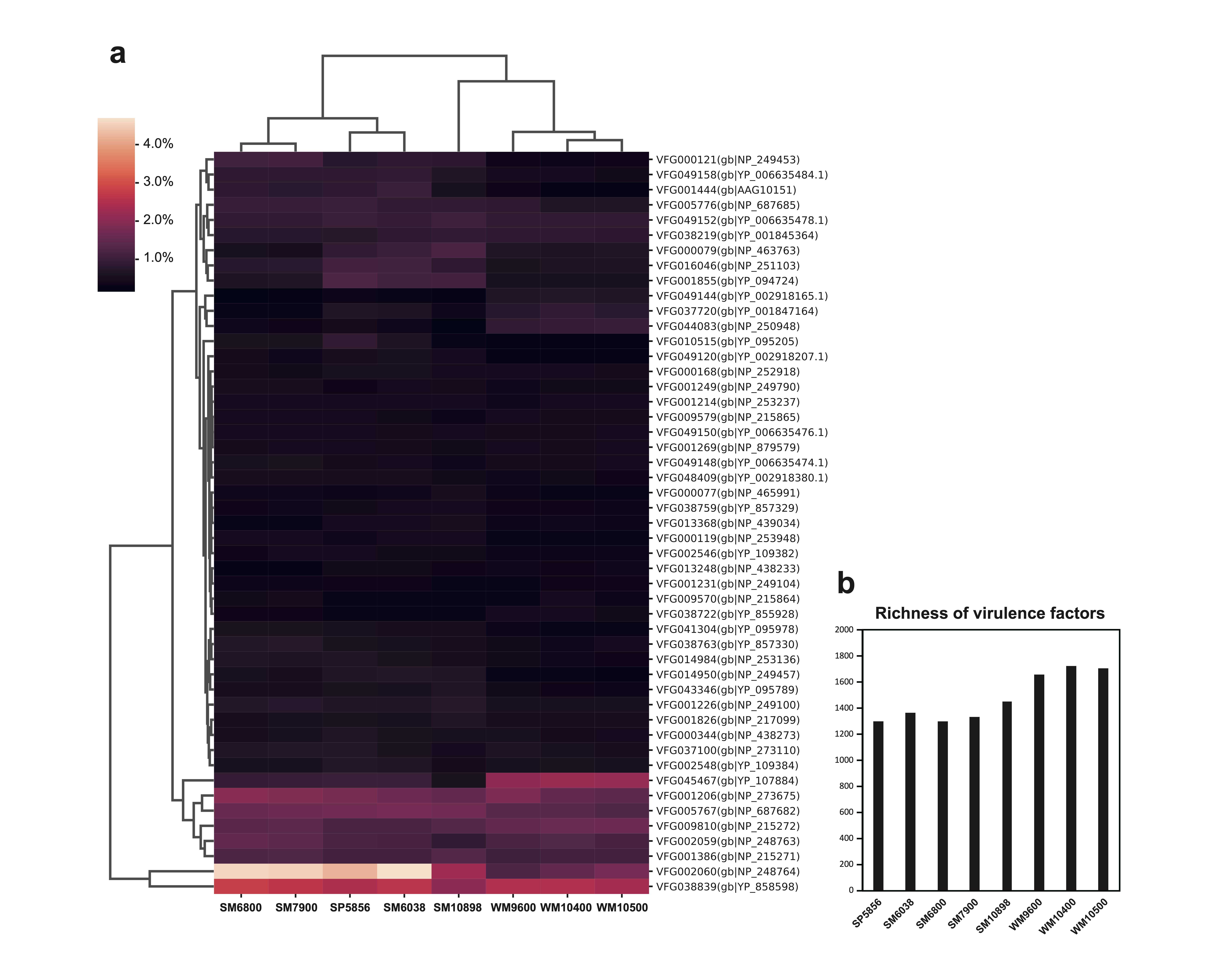


**Figure S3.** Relative abundance of virulence factor genes in the microbiome of the Mariana Trench and adjacent basin. 50 virulence factors with the highest abundance in these metagenomes were shown in (a). Color of each box is correlated with the relative abundance of each virulence factor in samples. (b) shows number of type of found virulence factors in each metagenome.





**Figure S4.** Relative abundance and richness of ARGs in the sediment microbiome of the Mariana Trench. The left Y axis and bar charts showed the relative percentage of ARG subtypes while the right Y axis and lines denoted the abundance sum (as copies per 16S rRNA gene copy, GP16S, b, c and d) of ARG subtypes. SP5856, SM6038 and SM10898 represented sediment metagenomes from different depths. The symbols before gene names, i.e., *, ** and !, denoted the degrees of fold change in abundance from SM10898 to SP5856 of ≥1, ≥10 and positive infinity, respectively.


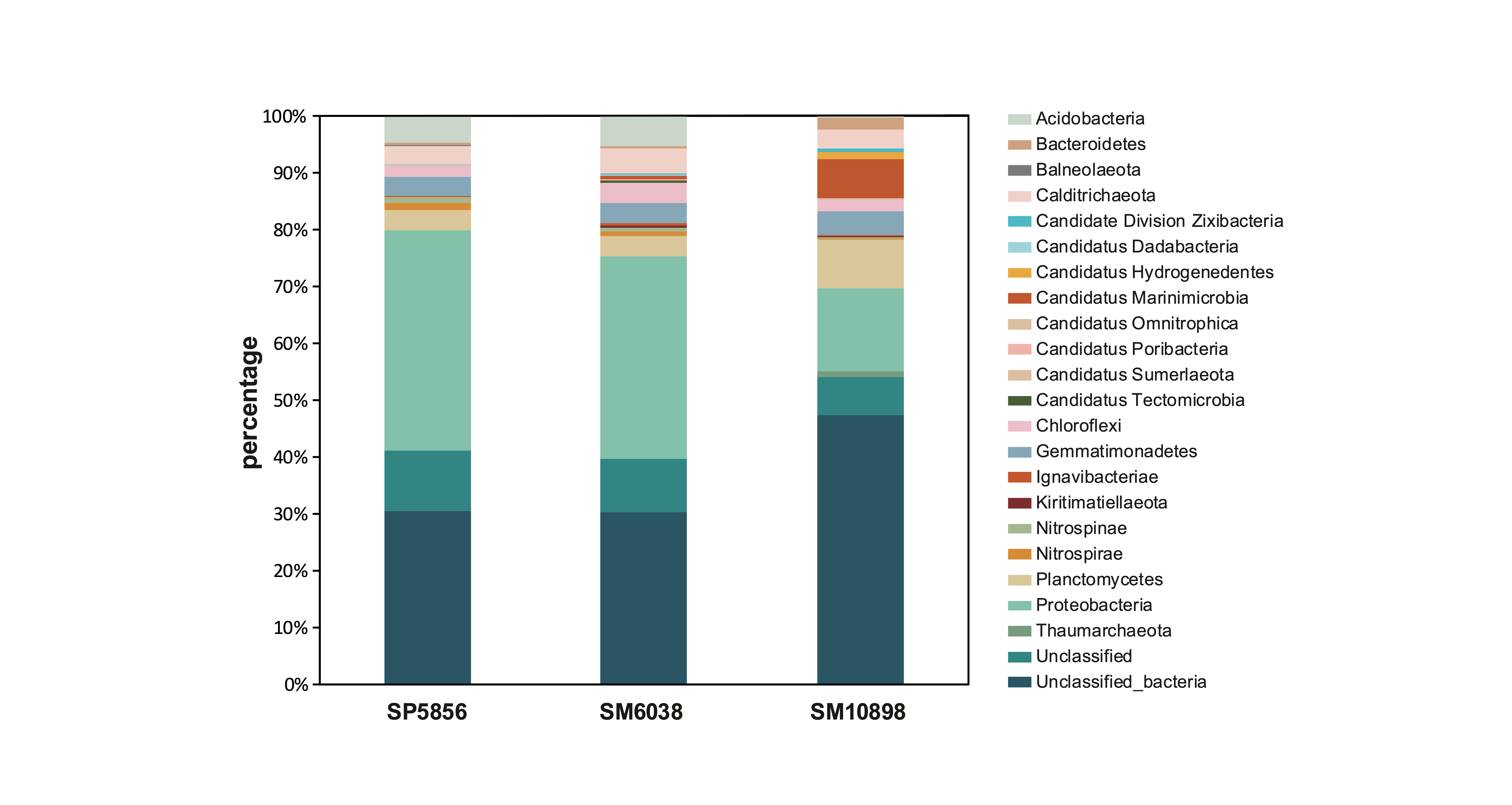


**Figure S5.** Relative distribution of the taxonomy (in phylum-level) of toxin gene-carrying resistance contigs taxonomy.


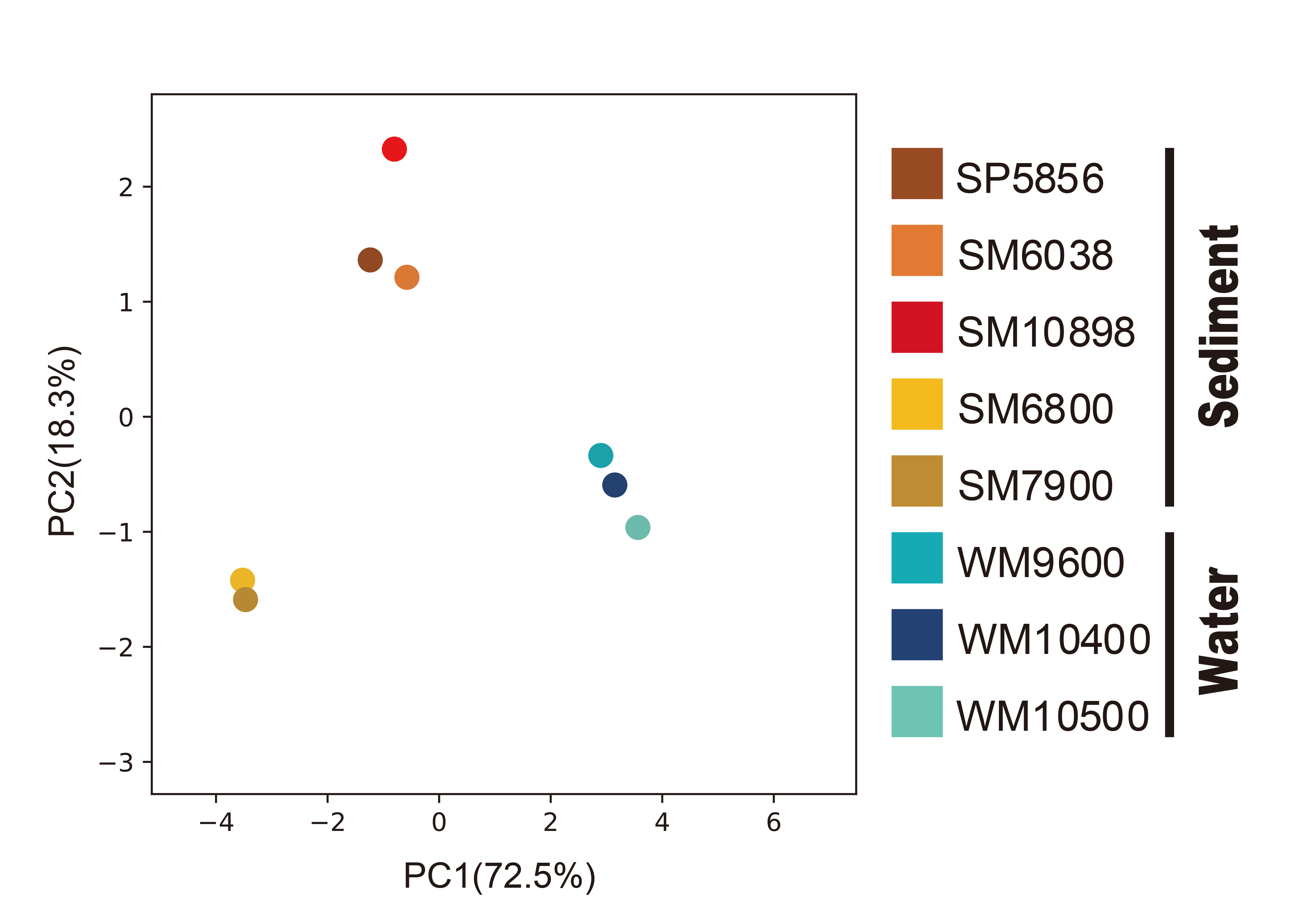


**Figure S6.** Principal component analysis of relative abundance of ARGs which was annotation by predicted ORFs, % variance explained shown in parentheses. SP5856, SM6038 and SM10898 represented sediment metagenomes from different depths. WM9600, WM10400, WM10500 represented seawater metagenomes from different depths.
